# Changes in Cannabis-Attributable Hospitalizations Following Nonmedical Cannabis Legalization in Canada

**DOI:** 10.1001/jamanetworkopen.2023.36113

**Published:** 2023-10-05

**Authors:** Daniel T. Myran, Adrienne Gaudreault, Lauren Konikoff, Robert Talarico, Rosalie Liccardo Pacula

**Affiliations:** 1 Bruyère Research Institute, Ottawa, Ontario, Canada; 2 Clinical Epidemiology Program, Ottawa Hospital Research Institute, Ottawa, Ontario, Canada; 3 Department of Family Medicine, University of Ottawa, Ottawa, Ontario, Canada; 4 ICES uOttawa, Ottawa Hospital Research Institute, Ottawa, Ontario, Canada; 5 Suzanne Dworak-Peck School of Social Work, University of Southern California, Los Angeles; 6 Institute for Addiction Science, University of Southern California, Los Angeles

## Abstract

**Importance:**

The impact of adult-use cannabis legalization and subsequent commercialization (ie, increasing store and product access) on hospitalizations in Canada is unclear.

**Objectives:**

To examine changes in overall and subtypes of hospitalizations due to cannabis and associated factors following legalization in Canada and to compare changes between provinces.

**Design, Setting, and Participants:**

This repeated cross-sectional analysis included all acute hospitalizations for individuals aged 15 to 105 years in Canada’s 4 most populous provinces (Ontario, Quebec, Alberta and British Columbia, population 26.9 million individuals in 2018). Data were obtained from routinely collected health administrative databases. Immediate and gradual changes in the age- and sex-standardized rates of hospitalizations due to cannabis were compared using an interrupted time series design over 3 time periods: prelegalization (January 2015 to September 2018), legalization with product and store restrictions (October 2018 to February 2020), and commercialization, which overlapped with the COVID-19 pandemic (March 2020 to March 2021).

**Main Outcomes and Measures:**

Rates of hospitalizations due to cannabis per 100 000 individuals and per 1000 all-cause hospital admissions.

**Results:**

There were 105 203 hospitalizations due to cannabis over the 7-year study period, 69 192 of which (65.8%) were among male individuals, and 34 678 (33%) of which were among individuals aged 15 to 24 years. Overall, the age- and sex-standardized rate of hospitalizations increased 1.62 times between January 2015 (3.99 per 100 000 individuals) and March 2021 (6.46 per 100 000 individuals). The largest relative increase in hospitalizations was for cannabis-induced psychosis (rate ratio, 1.40; 95% CI, 1.34 to 1.47 during the commercialization period relative to the prelegalization period). Nationally, legalization with restrictions was associated with a gradual monthly decrease of −0.06 (95% CI −0.08 to −0.03) in hospitalizations due to cannabis per 100 000 individuals. Commercialization and the COVID-19 pandemic were associated with an immediate increase of 0.83 (95% CI, 0.30 to 1.30) hospitalizations due to cannabis per 100 000 individuals. There was provincial variation in changes, with provinces with less mature legal markets experiencing the greatest declines immediately following legalization.

**Conclusions and Relevance:**

This cross-sectional study found that legalization with restrictions was not associated with an increase in hospitalizations due to cannabis but commercialization was. The findings suggest that commercialization of cannabis may be associated with increases in cannabis-related health harms, including cannabis-induced psychosis.

## Key Points

**Question:**

How have rates of hospitalizations due to cannabis changed, nationally and between provinces, since nonmedical cannabis was legalized in Canada?

**Findings:**

In this cross-sectional study of 26.9 million individuals in 4 Canadian provinces, rates of cannabis-related hospitalizations declined immediately after legalization during a period of legal market immaturity and later increased during a period of legal market expansion and the COVID-19 pandemic.

**Meaning:**

The findings in this study caution that greater levels of legal cannabis market access may be associated with an increase in a variety of cannabis-attributable harms.

## Teaser

This cross-sectional study evaluates changes in cannabis-related hospitalizations following the legalization of nonmedical cannabis use in Canada.

## Introduction

Canada legalized nonmedical cannabis for adult use in October 2018.^1^ There is increasing recognition that regular and heavy cannabis use is associated with various adverse health concerns, including psychosis, increased anxiety, depression, altered brain functioning, and cannabis dependence and addiction.^2,3,4,5^ Cannabis use results in a large number of health system encounters, including emergency department (ED) visits and hospitalizations, and is the leading cause of hospitalizations for substance use in Canadian youth.^6^ ED visits due to cannabis have also been increasing rapidly over time in the US and Europe.^7,8^ However, the impact of nonmedical cannabis legalization on health care visits due to cannabis remains unclear. Several studies have investigated changes over time in ED visits due to cannabis in two provinces (Alberta or Ontario) in Canada following legalization.^9,10,11,12,13,14^ Two brief reports have also examined changes in hospitalizations due to cannabis—a potentially more severe and specific indicator of cannabis-related health harms—in Ontario and Quebec.^15,16^ Studies to date have used differing lengths of follow-up and not compared changes following legalization in health service use due to cannabis between provinces or at the national level. This is an important gap, as prior work has established substantial variation in key cannabis policies, including product types and store access.^1,17,18^ Consequently, limited information is available about how cannabis retail policies may be associated with changes in cannabis harms postlegalization. In addition, to date, studies on changes in health service use due to cannabis following legalization have not directly compared changes across different types of presentations (eg, changes in visits for psychosis vs visit for intoxication), which limits knowledge about the kinds of cannabis harms that may have increased the most following legalization.

Although all provinces in Canada were legally required to allow the sale of cannabis in October 2018, each province and territory were allowed to set their own regulations regarding how cannabis would be sold and the types of products that could be introduced.^1^ Initially, all provinces in Canada were only allowed to sell dried cannabis flower. In October 2019, cannabis producers were allowed to apply to begin selling new high-potency cannabis products, including edibles, vape pens, and concentrates. Most provinces allowed these products to come to market starting in early 2020. Notably, Quebec did not permit these products over concern of their potential appeal to children and youth.^19^ Provinces also took differing approaches to cannabis retail store access. Alberta had a rapid expansion of stores and legal sales immediately following legalization. In contrast, Ontario and British Columbia initially had very low cannabis sales and store access, followed by rapid growth beginning in early 2020. Quebec had the lowest per capita stores and sales throughout the study. See eTable 1 in Supplement 1 for further details on cannabis policy in Canada.

These different approaches to legalization provide an opportunity to explore how different regulatory approaches to cannabis markets may impact the overall burden on public health. This study had 3 objectives: to examine changes at the national level in hospitalizations due to cannabis following legalization using data from Canada’s 4 most populous provinces (Ontario, Quebec, Alberta, and British Columbia, which together comprise 86% of the population of Canada); to compare changes in hospitalizations across the 4 provinces and contextualize changes by variation in cannabis retail policy; and to compare changes in hospitalizations according to different causes of cannabis-related harms (eg, acute intoxication, abuse, dependence, withdrawal, poisoning, and psychosis) to determine whether there have been any changes in these conditions over the 3 distinct policy periods in Canada since legalization.

### Methods

This repeated cross-sectional study was approved by the research ethics board of the Ottawa Hospital Research Institute. Because this study used deidentified aggregate health information, no informed consent was required. This study followed the Strengthening the Reporting of Observational Studies in Epidemiology (STROBE) reporting guideline.

### Study Design

We conducted a repeated cross-sectional population-based study using health administrative databases from Ontario, Quebec, Alberta, and British Columbia (population of 26.9 million individuals aged 15 to 105 years in 2018).^20^ We included hospitalizations from all individuals aged 15 to 105 years who were eligible for the province’s single-payer universal health insurance between January 2015 and March 2021. We calculated crude rates and age- and sex-standardized rates using direct standardization from the 2016 Canadian Census. We used an interrupted time series design to examine immediate and gradual changes in hospitalizations over 3 distinct policy periods: prelegalization (January 2015 to September 2018), legalization with strict controls (October 2018 to February 2020), and commercialization, including increasing store access and product type (March 2020 to March 2021), which overlapped with the COVID-19 pandemic. We began our study in 2015, as this was the start of hospitalization data being available in all 4 provinces. We categorized the transition between legalization with restrictions to commercialization as occurring in March 2020. This transition is consistent with prior work and reflects when expanded products became widely available (ie, mid-January 2020) and when store growth began to accelerate in Ontario (April 2020) in addition to the declaration of states of emergency across Canada (March 2020) along with large changes in health service use in response to the COVID-19 pandemic.^9,10,11,21^ As a sensitivity analysis, we defined the commercialization period as beginning in January 2020.

### Data Sources

Hospitalization data were obtained from the Canadian Institute of Health Informatics using the Discharge Abstract Database and the Hospital Morbidity Database between January 1, 2015, and September 30, 2021. These databases capture all acute care hospitalizations in Canada from each province’s universal health care insurance. An estimated 97% of Canadians are eligible for and covered by their province’s universal health coverage.^22^

### Outcomes

The primary outcome was the rate of hospitalizations due to cannabis per capita 100 000 per capita and per 1000 all-cause hospital admissions. We examined hospitalizations due to cannabis per 1000 all-cause hospitalizations to contextualize changes during the commercialization period with overall changes in health care visits. Hospitalizations due to cannabis were identified when 1 of the following *International Classification of Diseases*, *10th Revision* (*ICD-10*) codes was listed as the main or contributing reason for hospitalization: T40.7 (poisoning by cannabis, including derivatives) and F12.X (mental and behavioral disorders due to use of cannabinoids). We examined the monthly count of overall hospitalizations and subgroups, including sex and age (15 to 24 years and ≥25 years). We examined specific diagnostic types classified as acute intoxication (F12.0), harmful cannabis use (formerly cannabis abuse, F12.1), cannabis dependence (F12.2), cannabis withdrawal (F12.3), cannabis-induced psychosis (F12.5, F12.7), poisoning from cannabis and derivates (T40.7), and other or unspecified mental and behavioral disorders from cannabis (F12.6, F12.8, F12.9).

### Statistical Analysis

We present descriptive statistics to characterize and compare the absolute numbers and rate per person-years of hospitalizations due to cannabis across the 3 policy periods and the whole study period. We computed crude rate ratios with Wald 95% CIs.

We then calculated the monthly age- and sex-standardized rate of our primary outcomes per 100 000 individuals and per 1000 all-cause admissions over the 3 periods (prelegalization, legalization with restrictions, and commercialization/COVID-19). Rates were standardized using direct standardization with the 2016 Canadian Census as the reference population. We then used an interrupted time series approach with segmented linear regression analysis to assess the immediate and gradual changes in rates over the 3 policy periods. We included indicators representing the 4 seasons to account for seasonal variation, and all analyses included first-order autoregressive covariance structures. When examining rates per 100 000 individuals, we included 2 binary indicator variables, 1 for March 2020 and 1 for April 2020, to account for large decreases in overall health service use at onset of the pandemic, consistent with prior work. Models were estimated using restricted maximum likelihood.^23^ When visualizing monthly trends, we presented deseasonalized rates with the season fixed in winter. We expressed the immediate and gradual changes postlegalization as absolute mean rate changes with 95% CIs and interpreted statistical significance when the 95% CIs did not cross 1. All analyses were completed using SAS version 9.4 (SAS institute).

## Results

During the 7-year study period, there were 105 203 hospitalizations due to cannabis (63.36 per 100 000 person years), with 69 192 (65.8%) hospitalizations among male individuals and 34 678 (33.0%) among individuals aged 15 to 24 years. Table 1 lists the characteristics of hospitalizations due to cannabis for the whole study period, the prelegalization period, the legalization period, and the commercialization/COVID-19 pandemic period. The most common reason for admission was harmful cannabis use (51 631 of 105 203 [46.2%]), followed by cannabis dependence (22 266 of 105 203 [19.9%]), other (18 756 of 105 203 [16.8%]), and cannabis-induced psychosis (3387 of 105 203 [9.7%]). The largest relative inhcrease in hospitalization rates was for cannabis-induced psychosis (rate ratio, 1.40; 95% CI, 1.34 to 1.47 in the commercialization/COVID-19 period relative to prelegalization) and for cannabis withdrawal (rate ratio, 1.37; 95% CI, 1.20 to 1.56 in the legalization period relative to prelegalization). The largest absolute increase in hospitalization rates was for harmful cannabis use (35.16 during commercialization vs 29.15 prelegalization per 100 000 person years). Increases in hospitalizations due to cannabis over time were similar for men and women and greater for individuals 25 years and older compared to those aged 15 to 24 years.

**Table 1.** Characteristics of Individuals Hospitalized for Cannabis Use, by Province and by Reason for Admission From January 2015 to March 2021

Time period, characteristic	No. (%; rate per 100 000 person-y)				Rate ratio (95% CI)	
	Whole study (January 2015-March 2021)	Prelegalization (January 2015-September 2018)	Legalization (October 2018-February 2020)	Commercialization (March 2020-March 2021)	Legalization vs prelegalization	Commercialization vs prelegalization
National						
Overall	105 203 (63.36; NA)	59 117 (59.94; NA)	24 884 (64.29)	21 212 (70.50)	1.07 (1.05-1.09)	1.18 (1.16-1.20)
Sex						
Male	69 192 (65.77; 83.71)	38 946 (65.89; 80.03)	16 412 (65.95; 85.82)	13 834 (65.22; 93.05)	1.07 (1.05-1.09)	1.16 (1.14-1.18)
Female	36 011 (34.23; 42.48)	20 161 (34.11; 40.35)	8472 (34.05; 43.26)	7378 (34.78; 48.48)	1.07 (1.04-1.10)	1.20 (1.17-1.23)
Age, y						
15-24	34 678 (32.96; 141.89)	20 502 (34.69; 140.88)	7934 (31.88; 141.39)	6242 (29.43; 145.97)	1.00 (0.97-1.03)	1.04 (1.01-1.07)
≥25	70 525 (67.04; 49.33)	38 605 (65.31; 45.92)	16 950 (68.12; 51.22)	14 970 (70.57; 58)	1.12 (1.10-1.14)	1.26 (1.24-1.28)
Province						
Alberta	16 885 (16.04; 77.62)	9519 (16.10; 74.19)	3953 (15.88; 78.78)	3413 (16.09; 87.41)	1.06 (1.02-1.10)	1.18 (1.13-1.23)
British Columbia	26 570 (25.25; 99.52)	16 485 (27.88; 105.11)	5529 (22.21; 89.27)	4556 (21.48; 94.49)	0.85 (0.82-0.88)	0.90 (0.87-0.93)
Quebec	46 038 (43.76; 104.4)	24 384 (41.25; 93.31)	11 635 (46.76; 114.84)	10 019 (47.23; 127.91)	1.23 (1.20-1.26)	1.37 (1.34-1.40)
Ontario	15 710 (14.93; 20.98)	8719 (14.75; 19.82)	3767 (15.14; 21.7)	3224 (15.67; 23.83)	1.09 (1.05-1.13)	1.20 (1.15-1.23)
*ICD-10* diagnosis						
Acute intoxication	3438 (3.08; 2.07)	1936 (3.09; 1.96)	832 (3.15; 2.15)	670 (2.96; 2.23)	1.10 (1.01-1.19)	1.13 (1.03-1.23)
Harmful use	51 631 (46.20; 31.09)	28 752 (45.85; 29.15)	12 302 (46.58; 31.78)	10 577 (46.7; 35.16)	1.09 (1.07-1.11)	1.21 (1.18-1.24)
Dependence	22 266 (19.92; 13.41)	12 994 (20.72; 13.17)	5169 (19.57; 13.35)	4103 (18.11; 13.64)	1.01 (0.98-1.04)	1.04 (1.00-1.08)
Withdrawal	1444 (1.29; 0.87)	763 (1.22; 0.77)	363 (1.37; 0.94)	318 (1.40; 1.06)	1.21 (1.07-1.37)	1.37 (1.20-1.56)
Poisoning	3387 (3.03; 2.04)	1917 (3.06; 1.94)	828 (3.13; 2.14)	642 (2.83; 2.13)	1.10 (1.01-1.19)	1.10 (1.01-1.20)
Psychosis	10 845 (9.70; 6.53)	5828 (9.29; 5.91)	2520 (9.54; 6.51)	2497 (11.02; 8.3)	1.10 (1.05-1.15)	1.40 (1.34-1.47)
Other	18 756 (16.78; 11.3)	10 513 (16.77; 10.66)	4399 (16.65; 11.37)	3844 (16.97; 12.78)	1.07 (1.03-1.11)	1.20 (1.16-1.25)

Abbreviation: *ICD-10*, *International Classification of Diseases, 10th Revision*.

**Figure 1.**
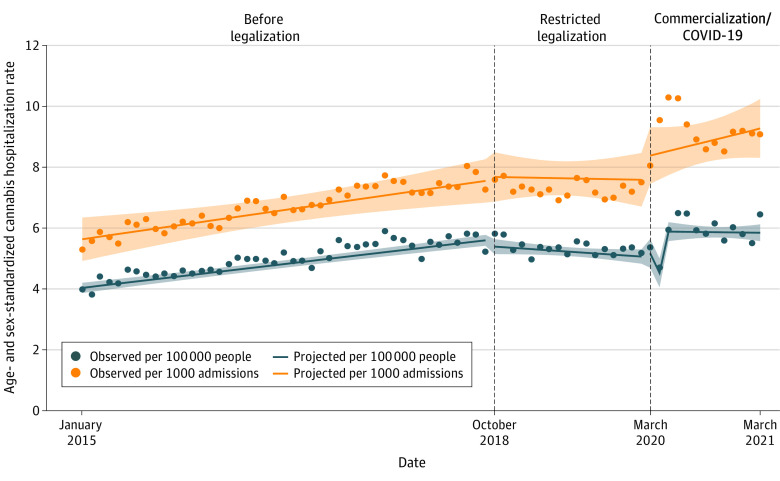
Age and Sex Standardized Rates of Hospitalizations Due to Cannabis for All of Canada Age and sex standardized rate of hospitalizations due to cannabis per 100 000 individuals and per 1000 all-cause hospital admissions between January 2015 and March 2021. Shaded regions indicate 95% CIs.

Figure 1 displays the national (4 combined provinces) age- and sex-standardized monthly rate of hospitalizations between January 2015 and March 2021 per 100 000 individuals and per 1000 hospitalizations due to cannabis. Between the first and last month of the study, the age- and sex-standardized rate of hospitalizations due to cannabis per 100 000 individuals increased 1.62 times from 3.99 per 100 000 individuals in January 2015 to 6.46 per 100 000 individuals in March 2021. Over the same period, the rate of hospitalizations due to cannabis per 1000 all-cause hospitalizations increased 1.71 times from 5.31 per 1000 hospitalizations in January 2015 to 9.10 per 1000 hospitalizations in March 2021.

Table 2 reports the segmented regression model coefficients for the absolute change in rates of monthly hospitalizations per capita nationally and for each of the 4 provinces. Nationally, the prelegalization age- and sex-standardized rates were increasing by 0.04 (95% CI, 0.03 to 0.04) hospitalizations per 100 000 individuals each month. The restricted legalization period was associated with an insignificant immediate level decrease of 0.19 (95% CI, −0.46 to 0.10) hospitalizations per 100 000 individuals per month and a significant monthly slope decrease of −0.06 (95% CI, −0.08 to −0.03) hospitalizations per 100 000 individuals. Cannabis commercialization and the overlapping COVID-19 period were associated with a significant immediate level change of 0.83 (95% CI, 0.36 to 1.30) hospitalizations per 100 000 individuals per month and an insignificant monthly slope increase of 0.02 (95% CI, −0.04 to 0.07) hospitalizations per 100 000 individuals. Similar patterns were observed for changes in visits per 1000 total hospital admissions, but the attenuation of the prelegalization trend during the restricted legalization period was no longer significant (see Table 2 for model coefficients for changes per 1000 admissions). Our sensitivity analysis starting the commercialization period during January 2020 showed a smaller immediate level change of 0.44 (95% CI, 0.03 to 0.84) monthly hospitalizations per 100 000 individuals and a greater monthly slope increase or 0.07 (95% CI, 0.02 to 0.12) hospitalizations per 100 000 individuals (see eFigure 1 and eTable 2 in Supplement 1 for changes over time and model coefficients).

**Table 2.** Change in Rates of Monthly Hospitalizations Due to Cannabis per Capita Overall and for Each Province, Following Nonmedical Cannabis Legalization and Commercialization and the COVID-19 Pandemic

Measure	Age- and sex-standardized monthly rate per 100 000 individuals (95% CI)				
	Canada	Alberta	British Columbia	Quebec	Ontario
Intercept (rate in January 2015)	4.01	5.55	7.95	5.41	1.28
Prelegalization monthly slope	0.04 (0.03 to 0.04)	0.02 (0.00 to 0.03)	0.02 (0.00 to 0.04)	0.10 (0.08 to 0.11)	0.01 (0.01 to 0.02)
Legalization with restrictions					
Legalization level change	−0.19 (−0.47 to 0.10)	0.33 (−0.56 to 1.23)	−0.43 (−1.36 to 0.51)	−0.43 (−0.96 to 0.11)	0.00 (−0.18 to 0.19)
Legalization monthly slope change	−0.06 (−0.08 to −0.03)	−0.04 (−0.12 to 0.04)	−0.16 (−0.24 to −0.07)	−0.07 (−0.12 to −0.02)	−0.02 (−0.04 to 0.00)
Legalization monthly slope	−0.02 (−0.04 to 0.00)	−0.03 (−0.10 to 0.05)	−0.14 (−0.22 to −0.06)	0.03 (−0.02 to 0.07)	−0.01 (−0.02 to 0.01)
Commercialization/COVID-19					
Commercialization/COVID-19 level change	0.83 (0.36 to 1.30)	1.78 (0.30 to 3.26)	1.67 (0.13 to 3.21)	0.55 (−0.34 to 1.45)	0.38 (0.0 to 0.69)
Commercialization/COVID-19 monthly slope change	0.02 (−0.04 to 0.07)	−0.06 (−0.23 to 0.11)	0.14 (−0.03 to 0.31)	0.02 (−0.08 to 0.13)	0.00 (−0.03 to 0.03)
Commercialization/COVID-19 monthly slope	0.00 (−0.05 to 0.04)	−0.09 (−0.23 to 0.06)	0.00 (−0.15 to 0.15)	0.05 (−0.04 to 0.14)	−0.01 (−0.04 to 0.02)
Age- and sex-standardized monthly rate per 1000 all-cause hospitalizations (95% CI)					
Intercept (rate in January 2015)	5.59	11.05	16.12	13.43	4.39
Prelegalization monthly slope	0.04 (0.02 to 0.07)	0.05 (0.00 to 0.10)	0.04 (0.00 to 0.08)	0.16 (0.13-0.19)	0.03 (0.02 to 0.04)
Legalization with restrictions					
Legalization level change	0.14 (−0.56 to 0.84)	1.72 (−0.59 to 4.02)	−0.92 (−2.73 to 0.89)	−0.36 (−1.83 to 1.11)	−0.20 (−0.81to 0.40)
Legalization monthly slope change	−0.05 (−0.14 to 0.04)	−0.15 (−0.36 to 0.06)	−0.23 (−0.39 to −0.07)	−0.10 (−0.23 to 0.02)	−0.07 (−0.12 to −0.01)
Legalization monthly slope	0.03 (−0.02 to 0.07)	−0.10 (−0.29 to 0.10)	0.19 (−0.34 to −0.03)	0.06 (−0.07 to 0.18)	−0.04 (−0.09 to 0.02)
Commercialization/COVID-19					
Commercialization/COVID-19 level change	0.72 (−0.04 to 1.49)	2.62 (−0.23 to 5.47)	2.93 (0.67 to 5.20)	4.95 (3.02-6.88)	1.71 (0.92 to 2.51)
Commercialization/COVID-19 monthly slope change	0.08 (−0.08 to 0.24)	0.18 (−0.19 to 0.56)	0.27 (−0.0 to 0.57)	−0.12 (−0.35 to 0.11)	0.01 (−0.08 to 0.11)
Commercialization/COVID-19 monthly slope	0.05 (−0.04 to 0.14)	0.08 (−0.21 to 0.38)	0.09 (−0.14 to 0.32)	−0.06 (−0.25 to 0.13)	−0.02 (−0.10 to 0.06)

**Figure 2.**
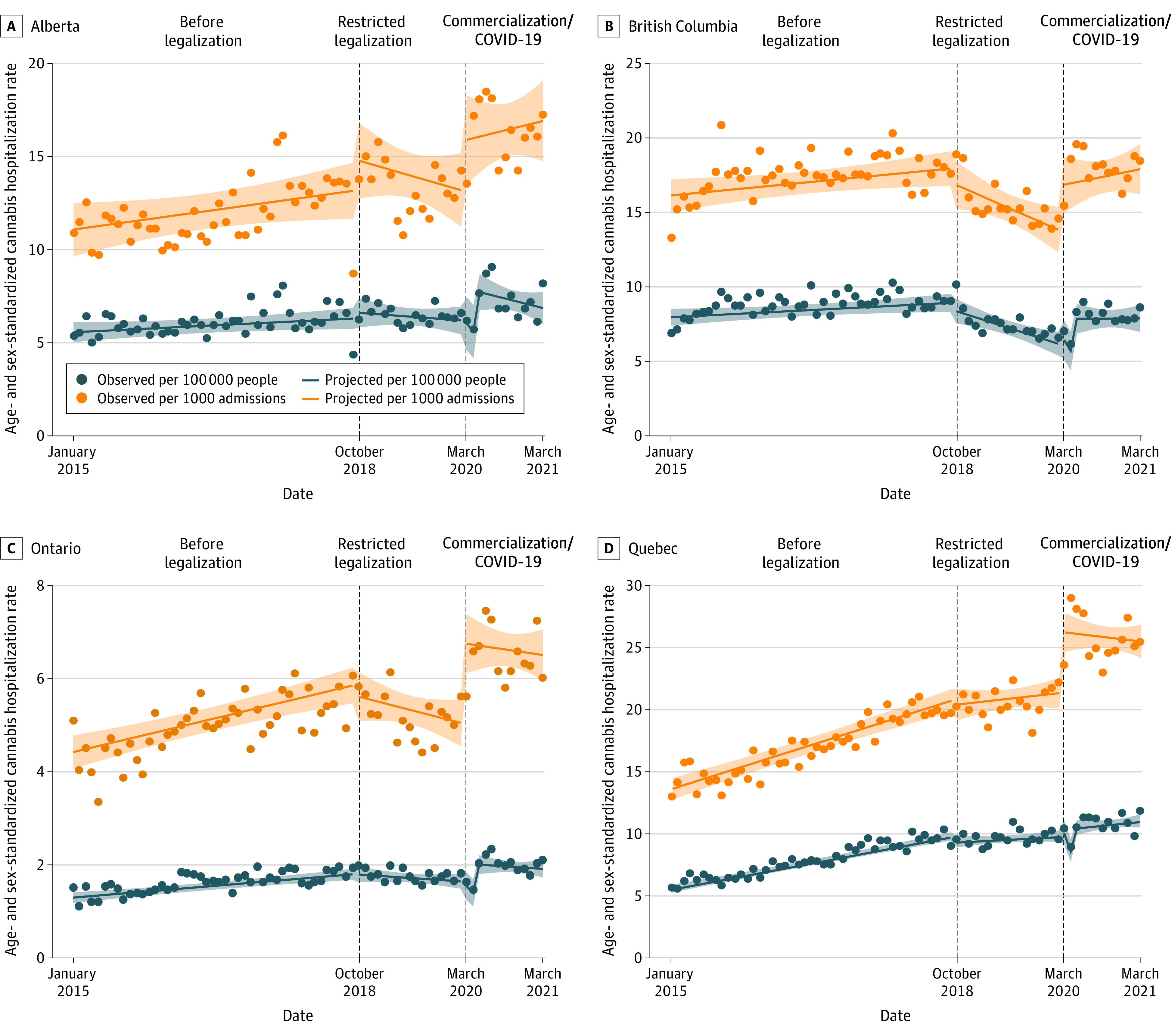
Age and Sex Standardized Rates of Hospitalizations Due to Cannabis for Each Province Age and sex standardized rate of hospitalizations due to cannabis per 100 000 individuals and per 1000 all-cause hospital admissions between January 2015 and March 2021 for each province. Shaded regions indicate 95% CIs.

Figure 2 displays monthly age- and sex-standardized rates of hospitalizations between January 2015 and March 2021 per 100 000 and 1000 admissions separately for each of the four provinces. Quebec had the largest increase in hospitalizations, from 5.71 per 100 000 individuals in January 2015 to 11.84 per 100 000 individuals in March 2021. Alberta had the second largest increase, from 5.40 in January 2015 to 8.21 in March 2021, followed by Ontario (1.51 per 100 000 individuals in January 2015 to 2.01 per 100 000 individuals in Mach 2021), and British Columbia experienced the smallest increase in hospitalizations from 6.92 per 100 000 individuals in January 2015 to 8.21 per 100 000 individuals in March 2021. Prelegalization visits were increasing 10 times faster in Quebec than Ontario. The restricted legalization phase was associated with a significant attenuation in the prelegalization slope for all provinces except Alberta. Commercialization/COVID-19 were associated with a significant level increase in all provinces except Quebec but no significant slope change was observed in any province.

## Discussion

Over our 7-year study period, there was a 1.62-fold increase in the age- and sex-standardized rates of monthly hospitalizations due to cannabis in Canada. The first 1.5 years following legalization, when stores and products were restricted, was associated with a modest decrease in hospitalizations. In contrast, the period when cannabis became broadly commercialized in Canada, which overlapped with the COVID-19 pandemic, was associated with increased hospitalizations due to cannabis. Men had almost double the rates of hospitalizations due to cannabis throughout the study period, but this disparity was constant over time. Increases in cannabis-related hospitalizations were greater in individuals 25 years and older compared to those aged 15 to 24 years. There was variation in changes postlegalization in the type of hospitalization due to cannabis, with the largest relative increases for cannabis-induced psychosis. Broadly, these findings caution that greater levels of market commercialization and product proliferation may increase a variety of cannabis-attributable harms. However, the current study cannot isolate the impacts of the COVID-19 pandemic.

Changes in hospitalizations due to cannabis during our study period varied by regulatory period. Hospitalizations were increasing rapidly leading up to legalization, consistent with increasing cannabis use over time.^24^ Several factors may have contributed to this trend, including changing social norms related to cannabis use and increasing access from illicit nonmedical and legal medical sources.^25,26^ Hospitalizations decreased immediately following legalization, which may have been the result of decreased cannabis availability in the early phases of legalization due to national shortages of legal cannabis product and contraction in the illegal market (eg, increased enforcement activities and voluntary closures of illicit dispensaries while trying to open a legal store).^27,28^ Hospitalizations began increasing again in early 2020, coinciding with a major expansion of retail access, an increasing variety of highly potent legal cannabis products, and the COVID-19 pandemic.^29,30,31^ Disentangling COVID-19 from commercialization is challenging, but several factors support the theory that commercialization contributed to the observed increases. First, increases hospitalizations due to cannabis were far greater than all-cause hospitalizations and substance-use specific hospitalizations, suggesting that a factor other than pandemic influences on general substance use or health service use contributed.^32^ Second, our sensitivity analysis using a commercialization starting point prepandemic (January 2020) showed similar results, suggesting that hospitalizations due to cannabis began increasing before the onset of the pandemic. Third, higher-potency cannabis products, which are associated with increased risk of harm, increased during the commercialization period following the introduction of new high-potency products starting in January 2020 (eg, vape pens and concentrates), along with a general trend of increasing tetrahydrocannabinol content in legal dried flower products since legalization.^33,34^ The findings are also consistent with increases in ED visits and hospitalizations due to cannabis from US states that legalized nonmedical cannabis before the pandemic.^35,36,37^

We observed substantial variation in changes over time in hospitalizations due to cannabis by province in Canada. Hospitalizations increased in all provinces over time in the lead-up to legalization. During the restricted legalization period, hospitalizations began decreasing over time in Ontario and British Columbia, which is consistent with legal sales data showing Ontario ($1.48 CAD) and British Columbia ($ 0.95 CAD) had the lowest per capita legal cannabis sales (average monthly sales in $CAD per individual 15 years and older) during the first year after legalization. Visits declined to a lesser extent in Quebec and did not change over time in Alberta, consistent with these provinces having 2 to 4 times higher legal sales over the first year (Alberta, $4.61 CAD and Quebec $2.27 CAD).^38^ The greatest increases in hospitalizations due to cannabis during the commercialization/COVID-19 period were in Ontario and British Columbia, which had much larger legal cannabis sales growth between the final and first year of the study than Alberta and Quebec (see eFigure 2 in Supplement 1 for monthly cannabis sales over time). Our findings are also consistent with the prevalence of self-reported past 3-month cannabis use, which have not changed postlegalization in Quebec but have increased in Alberta, Ontario, and British Columbia.^39^ Ongoing monitoring postpandemic and as the market continues to expand is needed.

To date, most studies examining changes in ED visits or hospitalizations due to cannabis postlegalization in Canada have either examined a single type of cannabis health care visit (eg, cannabis-induced psychosis) or aggregated visit types together.^9,10,11,12,13,40^ Consequently, there is no data on direct comparisons on changes in different types of cannabis harms over time. We observed that cannabis-induced psychosis had the largest relative increase in rates of hospitalizations. Prior evidence suggests that one-third of individuals with first-presentation cannabis-induced psychosis will subsequently develop schizophrenia, raising concerns about potential important long-term impacts of legalization.^41^ The largest absolute increase in rates of hospitalization was for harmful cannabis use. These increases in cannabis-induced psychosis and harmful cannabis use (eg, cannabis use disorders) are consistent with reported increases in daily cannabis after legalization use in Canada following legalization along with the availability of higher-potency cannabis products both of which increase the risk of adverse events.^33,34,42^

Our findings have important policy implications within Canada and other countries considering legalizing cannabis. They suggest that cannabis legalization with strict controls may not cause any immediate change in rates of hospitalizations and may even have a modest public health benefit, as legalization in this study was associated with a decrease in cannabis-related health service use visits. However, our findings also caution that commercialization and rapid expansion of the cannabis retail market may reverse these benefits with consistent increases in hospitalizations due to cannabis across the different provincial regulatory approaches in Canada.^43^

### Limitations

This study has limitations. First, as previously discussed, the close timing of the COVID-19 pandemic and the cannabis retail market commercialization prevent causal attribution of either event to the observed changes.^32^ Second, because of coding changes (migration from *ICD-9* to *ICD-10* in April 2019) we excluded hospitalizations in specialized mental health hospitals from Ontario, the most populous province in Canada. Consequently, our study underestimates the population-level rate of hospitalizations due to cannabis in Ontario and when aggregated at the national level. Third, greater physician awareness and willingness of patients to disclose cannabis use after legalization could contribute to observed increases. However, this bias is unlikely to be responsible for changes observed in the study (eg, initial decline followed by an increase). Fourth, while the codes used to identify hospitalizations due to cannabis are part of an established indicator, further research is needed to understand their ability to differentiate specific presentations.^6^ Fifth, there were large differences in rates of hospitalizations due to cannabis between provinces which likely reflect both health system differences (eg, bed capacity) and differences in documentation of cannabis harms. While this variation does not challenge within-province comparisons, it does limit comparability between provinces. Sixth, use of aggregate health administrative data limited our ability to investigate important clinical questions (eg, whether increases were related to new cannabis use disorders or exacerbation of existing ones), and further research using alternative designs is indicated.

## Conclusions

In this cross-sectional study, hospitalizations due to cannabis were increasing prelegalization and may have increased further following the legalization of nonmedical cannabis use by adults in Canada. The findings suggest that the initial period of time following legalization in Canada with tightly controlled products and limited store access was not associated with increases in hospitalizations due to cannabis. In contrast, there is some evidence to support that the period in which stores and products expanded was associated with increases in cannabis hospitalizations, particularly for cannabis-induced psychosis. Importantly, the overlap of store and product expansion and the COVID-19 pandemic challenge attribution of increases. Ongoing surveillance efforts are needed, but restrictions on product type and store access may be reasonably precautionary measures for jurisdictions consideration legalization.

## Notes

Supplement 1. **eTable 1.** Timeline of legalization of cannabis in Canada **eTable 2.** Sensitivity analysis using different starting dates for commercialization for immediate and gradual changes in rates of monthly hospitalizations due to cannabis in four largest provinces in Canada **eTable 3.** Changes in cannabis sales in first and last year of study **eFigure 1.** Sensitivity analysis using January 2020 as start of commercialization period of age and sex standardized rates of hospitalizations due to cannabis per 100,000 individuals and per 1,000 all-cause hospitalizations due to cannabis in the four largest provinces in Canada **eFigure 2.** Changes over time in monthly per capita spending on cannabis ($CAD per individual aged 15+years) in Canada, Ontario, Alberta, Quebec and British Columbia
eReferences.

Supplement 2.
Data Sharing Statement
