# Supplementary material for: Changes in Cannabis-Attributable Hospitalizations Following Nonmedical Cannabis Legalization in Canada
Source: JAMA Netw Open. 2023 Oct 5;6(10):e2336113. doi: 10.1001/jamanetworkopen.2023.36113 (PMC10556968; doi:10.1001/jamanetworkopen.2023.36113)
Supplement: Supplement 1. — eTable 1. Timeline of legalization of cannabis in Canada eTable 2. Sensitivity analysis using different starting dates for commercialization for immediate and gradual changes in rates of monthly hospitalizations due to cannabis in four largest provinces in Canada eTable 3. Changes in cannabis sales in first and last year of study eFigure 1. Sensitivity analysis using January 2020 as start of commercialization period of age and sex standardized rates of hospitalizations due to cannabis per 100,000 individuals and per 1,000 all-cause hospitalizations due to cannabis in the four largest provinces in Canada eFigure 2. Changes over time in monthly per capita spending on cannabis ($CAD per individual aged 15+years) in Canada, Ontario, Alberta, Quebec and British Columbia eReferences. [file jamanetwopen-e2336113-s001.pdf]

Supplemental Online Content

Myran DT, Gaudreault A, Konikoff L, Talarico R, Liccardo Pacula R. Changes in cannabis-attributable hospitalizations following nonmedical cannabis legalization in Canada. *JAMA Netw Open*. 2023;6(10):e2336113.
doi:10.1001/jamanetworkopen.2023.36113

eTable 1. Timeline of legalization of cannabis in Canada
eTable 2. Sensitivity Analysis using different starting dates for commercialization for immediate and gradual changes in rates of monthly hospitalizations due to cannabis in four largest provinces in Canada
eTable 3. Changes in cannabis sales in first and last year of study
eFigure 1. Sensitivity analysis using January 2020 as start of commercialization period of age and sex standardized rates of hospitalizations due to cannabis per 100,000 individuals and per 1,000 all-cause hospitalizations due to cannabis in the four largest provinces in Canada
eFigure 2. Changes over time in monthly per capita spending on cannabis ($CAD per individual aged 15+years) in Canada, Ontario, Alberta, Quebec and British Columbia
eReferences

This supplemental material has been provided by the authors to give readers additional information about their work.

© 2023 Myran DT et al. *JAMA Network Open.*

eTable 1. Timeline of legalization of cannabis in Canada

Date	Policy Change	Description
**Legalization and liberalization of medical cannabis**		
July 2001	Medical cannabis legalized under the Marihuana Medical Access Regulations (MMAR)	Access to medical cannabis for a limited list of severe or chronic medical conditions. Applications are made directly to Health Canada. Most individuals receive an individual license to grow their own cannabis.(1)
April 2014	Liberalization of medical cannabis under the Marihuana for Medical Purposes Regulations (MMPR) regime	Access to medical cannabis is expanded for any individuals who received authorization from a physician or nurse practitioner stating that the individual will benefit from medical cannabis. Authorized individuals access cannabis from a commercial grower. Access to medical cannabis varies considerably by province with the highest access in British Columbia.(1)
December 2015	Government announcement of intention to legalize non-medical Cannabis	The Federal Government announces intentions to legalize non-medical cannabis.(2)
**Legalization of non-medical cannabis**		
October 2018	Legalization of non-medical cannabis under the Cannabis Act	Canadians of legal age can legally consume and possess cannabis. The legislation allowed provinces and territories to establish their own regulations for cannabis sales and distribution. Only the sale of dried cannabis flower, seeds and oil products permitted.(3)<br>Quebec sells cannabis through a fully public model with government operated stores, a government run online sales model, and an initial minimum legal age of purchase of 18 years which switches to 21 years in January 2020.<br>Alberta sells cannabis through a hybrid model with privately operated stores, public online sales (converted to private online sales in March 2022), and a minimum legal age of purchase of 18 years.<br>Ontario sells cannabis through a hybrid model with privately operating stores, a government run online sales model, and a minimum legal age of purchase of 19 years. Until April 1, 2019 no retail stores were permitted to open, after which there were strict limits on retail stores.(4)<br>British Columbia sells cannabis through a hybrid model with both private and government operated stores, a government run online sales model, and a minimum legal age of purchase of 19 years.

© 2023 Myran DT et al. *JAMA Network Open.*

Commercialization of non-medical cannabis/COVID-19 pandemic		
Late December/ January 2020	New high products begin to be sold	Sale of new products with high THC contents (concentrates, vapes, and commercially produced edibles) begin in all provinces except Quebec.(5)(6)
March 2020	COVID-19 Pandemic	The World Health Organization (WHO) declares COVID-19 a global pandemic
Variable	Expansion of cannabis store access	Store expansion differs by province. Largest expansion over time occurs in Ontario were government removes the cap on the number of stores in April 2020.(4)<br><br>At end of study (April 2021) the number of cannabis stores per 100,000 individuals 15+ (7) by province is:<br>Quebec: 0.93<br>Ontario: 4.79<br>British Columbia: 7.32<br>Alberta: 16.51

eTable 2. Sensitivity Analysis using different starting dates for commercialization for immediate and gradual changes in rates of monthly hospitalizations due to cannabis in four largest provinces in Canada

© 2023 Myran DT et al. *JAMA Network Open.*

Measure	Commercialization Starts March 2020	Commercialization Starts January 2020
	Standardized[A] Monthly Rate per 100,000 individuals (95%CI)	
Intercept (Rate in January 2015)	4.01	3.96
Pre Legalization Monthly Slope	0.04 (0.03-0.04)	0.04 (0.03-0.04)
**Legalization with restrictions**		
Legalization Level Change	-0.19 (-0.47 to 0.10)	-0.08 (-0.40 to 0.25)
Legalization Monthly Slope Change	-0.06 (-0.08 to -0.03)	-0.07 (-0.10 to -0.04)
Legalization Monthly Slope	-0.02 (-0.04 to 0.00)	-0.03 (-0.06 to 0.00)
**Commercialization/COVID-19**		
Commercialization/ COVID-19 Level Change	0.83 (0.36-1.30)	0.44 (0.03-0.84)
Commercialization / COVID-19 Monthly Slope Change	0.02 (-0.04 to 0.07)	0.07 (0.02-0.12)
Commercialization / COVID-19 Monthly Slope	0.00 (-0.05 to 0.04)	0.04 (0.01-0.07)
	Standardized[A] Monthly Rate per 1,000 overall hospitalizations (95%CI)	
Intercept (Rate in January 2015)	5.59	5.51
Pre Legalization Monthly Slope	0.04 (0.02-0.07)	0.05 (0.00-0.09)
**Legalization with restrictions**		
Legalization Level Change	0.14 (-0.56 to 0.84)	0.29 (-0.45 to 1.03)
Legalization Monthly Slope Change	-0.05 (-0.14 to 0.04)	-0.06 (-0.21 to 0.09)
Legalization Monthly Slope	0.03 (-0.02 to 0.07)	-0.01 (-0.13 to 0.11)
**Commercialization/COVID-19**		
Commercialization/ COVID-19 Level Change	0.72 (-0.04 to 1.49)	-0.24 (-0.98 to 0.50)
Commercialization / COVID-19 Monthly Slope Change	0.08 (-0.08 to 0.24)	0.14 (-0.07 to 0.36)
Commercialization / COVID-19 Monthly Slope	0.05 (-0.04 to 0.14)	0.13 (0.00-0.26)

[A]Age-sex standardized rate

© 2023 Myran DT et al. *JAMA Network Open.*

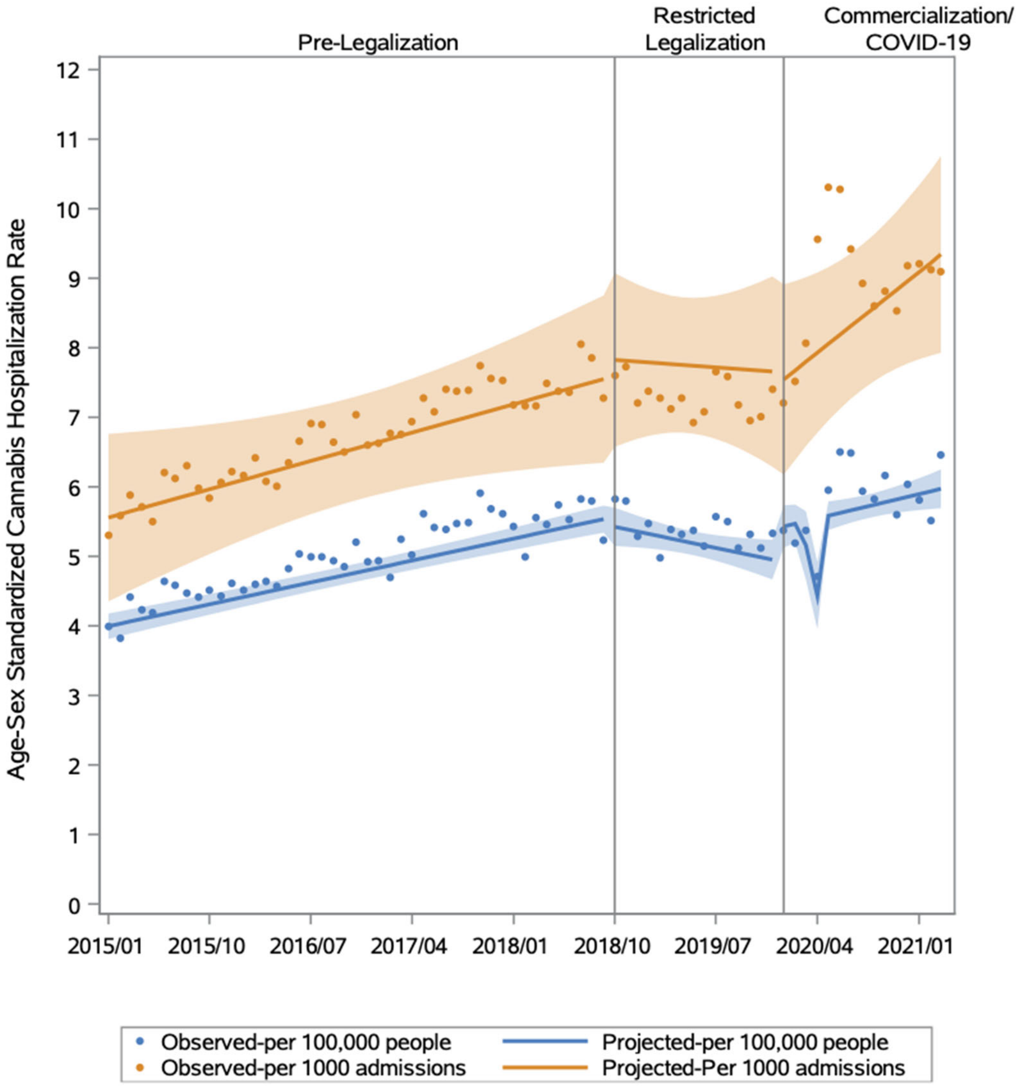

eFigure 1. Sensitivity analysis using January 2020 as start of commercialization period of age and sex standardized rates of hospitalizations due to cannabis per 100,000 individuals and per 1,000 all-cause hospitalizations due to cannabis in the four largest provinces in Canada

Shaded lines indicated 95% confidence intervals.

© 2023 Myran DT et al. *JAMA Network Open.*

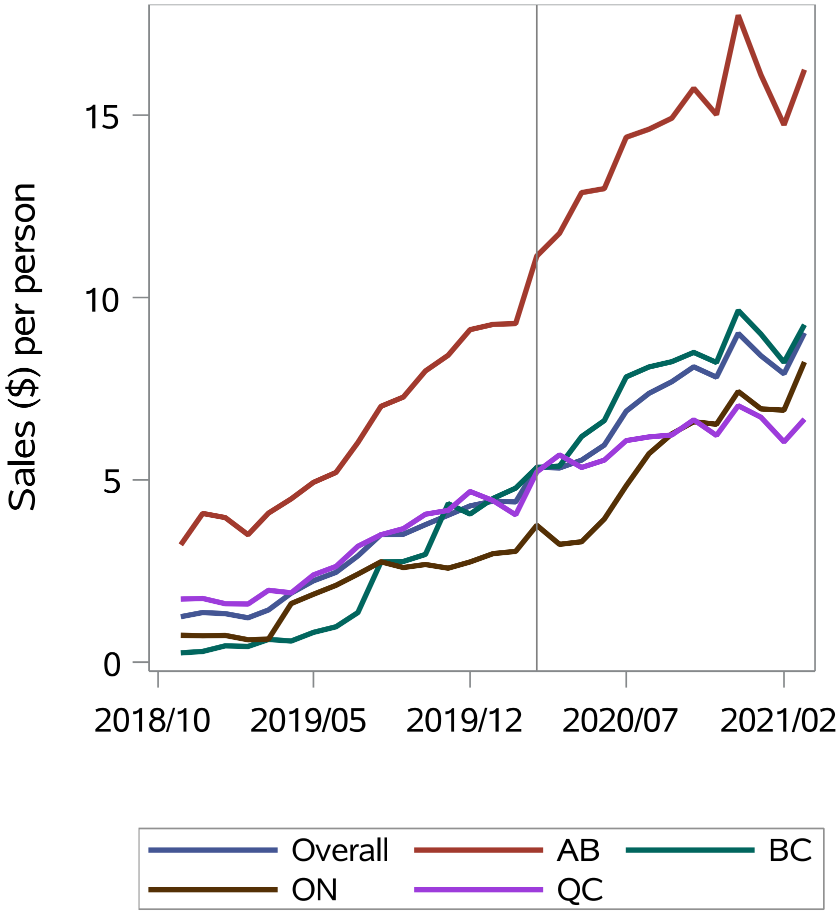

Sales ($) per person
0
5
10
15
2018/10
2019/05
2019/12
2020/07
2021/02
Overall
AB
BC
ON
QC

eTable 3. Changes in cannabis sales in first and last year of study

Province	British Columbia	Ontario	Alberta	Quebec
	Average Monthly Legal Sales Per Individual aged 15+ in CAD			
First Year of Study (October 2018 - September 2019)	0.95	1.48	4.61	2.27
Last Year of Study (April 2020 - March 2021)	7.93	5.82	14.76	6.20
Percent Change between first and last year of study	734.65	294.75	219.91	172.58

© 2023 Myran DT et al. *JAMA Network Open.*

eReferences

1. Shim M, Nguyen H, Grootendorst P. Lessons from 20 years of medical cannabis use in Canada. PLoS One. 2023 Mar 1;18(3):e0271079.
2. Task Force on Cannabis Legalization and Regulation. A Framework for the Legalization and Regulation of Cannabis in Canada. Health Canada. 2016; Available from: https://www.canada.ca/en/health-canada/services/drugs-medication/cannabis/laws-regulations/task-force-cannabis-legalization-regulation/framework-legalization-regulation-cannabis-in-canada.html
3. Parliament of Canada. Bill C-45: The Cannabis Act. 2018.
4. Myran DT, Pugliese M, Tanuseputro P, Cantor N, Rhodes E, Taljaard M. The association between recreational cannabis legalization, commercialization and cannabis-attributable emergency department visits in Ontario, Canada: an interrupted time–series analysis. Addiction. 2022
5. Canada Gazette, Part 2, Volume 153, Number 13: Regulations Amending the Cannabis Regulations (New Classes of Cannabis). [cited 2023 Aug 14]. Available from: https://gazette.gc.ca/rp-pr/p2/2019/2019-06-26/html/sor-dors206-eng.html
6. CQLR c C-5.3, r.0.1 | Regulation to determine other classes of cannabis that may be sold by the Société québécoise du cannabis and certain standards respecting the composition and characteristics of cannabis.
7. Myran DT, Friesen EL, Dickson S, Konikoff L, Arora G, Tanuseputro P. Access to legal cannabis market in Canada over the four years following non-medical cannabis legalisation. Drug Alcohol Rev. 2023;

© 2023 Myran DT et al. *JAMA Network Open.*
