# Supplementary material for: Changes in Cannabis-Attributable Hospitalizations Following Nonmedical Cannabis Legalization in Canada
Source: JAMA Netw Open. 2023 Oct 5;6(10):e2336113. doi: 10.1001/jamanetworkopen.2023.36113 (PMC10556968; doi:10.1001/jamanetworkopen.2023.36113)
Supplement: Supplement 2. — Data Sharing Statement [file jamanetwopen-e2336113-s002.pdf]

Data Sharing Statement

Myran. Changes in Cannabis-Attributable Hospitalizations Following Nonmedical Cannabis Legalization in Canada. *JAMA Netw Open*. Published October 05, 2023.
doi:10.1001/jamanetworkopen.2023.36113

Data
Data available: No

Additional Information
Explanation for why data not available: The data set from this study is held securely at the Institute for Clinical Evaluative Sciences (ICES). While data-sharing agreements prohibit ICES from making the data set publicly available, access may be granted to those who meet pre-specified criteria for confidential access, available at https://www.ices.on.ca/DAS.
